# Beyond climate: the emerging physiological roles of methane and nitrous oxide

**DOI:** 10.4103/mgr.MEDGASRES-D-25-00242

**Published:** 2026-01-15

**Authors:** Frank Keppler, Daniela Polag, Moritz Schroll, Mihaly Boros

**Affiliations:** Institute of Earth Sciences, Heidelberg University, Heidelberg, Germany; Heidelberg Center for the Environment (HCE), Heidelberg University, Heidelberg, Germany; Institute of Surgical Research, University of Szeged, Szeged, Hungary

**Figure 1:**
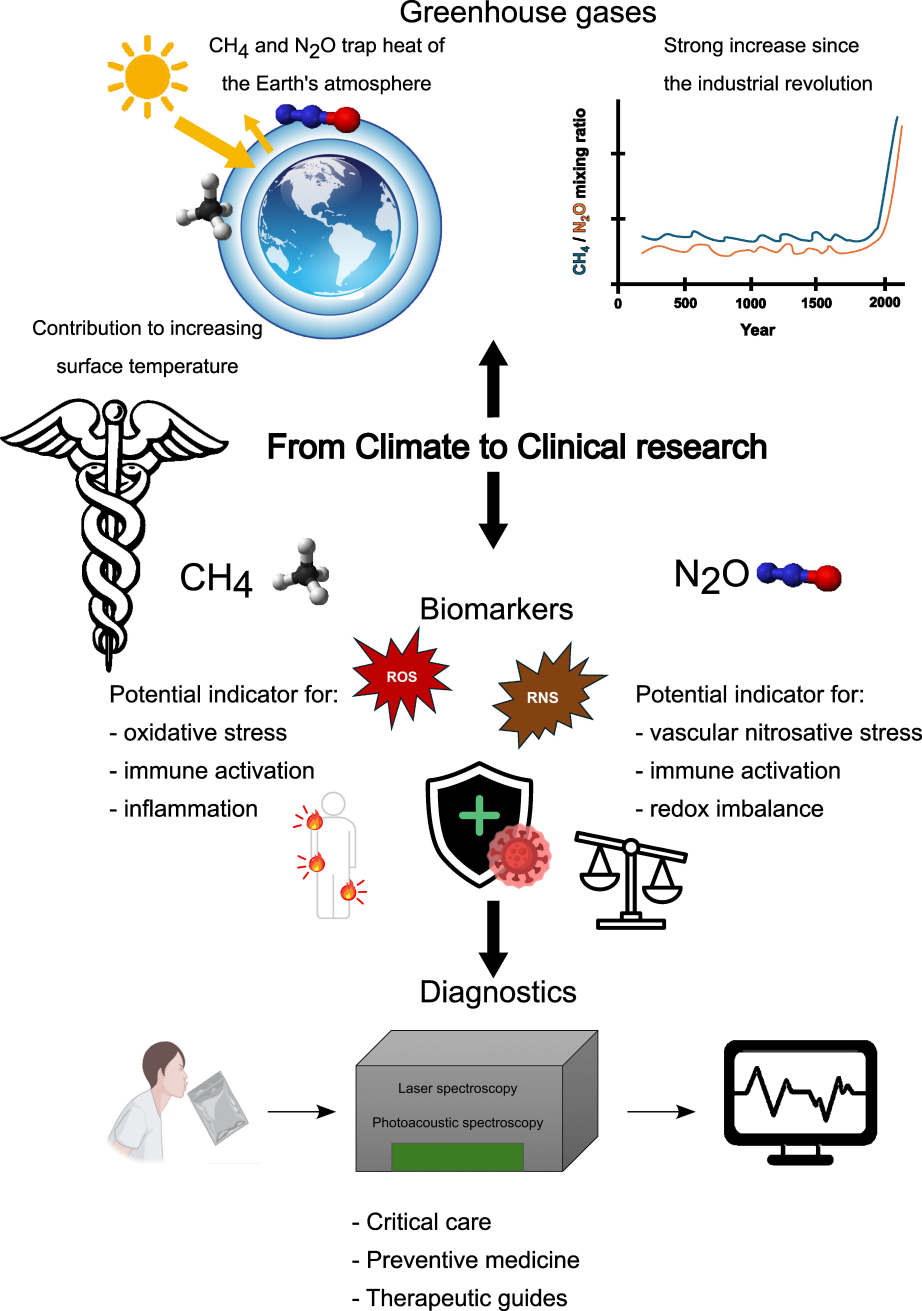
The dual role of methane (CH_4_) and nitrous oxide (N_2_O): from climate to clinic. Upper panel: Their established importance as greenhouse gases in Earth sciences. Lower panel: Their emerging potential in medicine as biomarkers of oxidative and nitrosative stress, detectable through non-invasive breath analysis, such as laser and photoacoustic spectroscopy. Beyond reflecting systemic redox balance, CH_4_ and N_2_O show promise as diagnostic tools for conditions such as inflammatory bowel disease, rheumatoid arthritis, and gastrointestinal ischemia. In critical care, continuous monitoring may provide early warning of redox imbalances during surgery, sepsis, or extracorporeal support. In preventive medicine, individual baseline profiles could reveal subtle physiological changes before symptoms occur. Both gases may also serve as therapeutic guides, with breath analysis offering real-time feedback on interventions such as antioxidant therapy, dietary nitrate modulation, or iron chelation. Created with Canva (https://www.canva.com) and Inkscape (version 1.3.2; https://inkscape.org). RNS: Reactive nitrogen species; ROS: reactive oxygen species.

**Methane (CH_4_) and nitrous oxide (N_2_O) beyond climate change:** CH_4_ and N_2_O are among the most potent greenhouse gases, exerting strong effects on climate and atmospheric chemistry. In Earth system science, their roles and the biogeochemical pathways that generate them have been studied for decades.^1^,^2^ Traditionally, biological CH_4_ production was attributed almost exclusively to anaerobic methanogenesis in wetlands, ruminants, and other anoxic environments, whereas N_2_O was viewed as a byproduct of microbial nitrification and denitrification in soils. In human physiology, these gases were long considered relevant only in the context of environmental exposure or as byproducts of microbial activity in the gut or oral cavity. This perception is now changing. Multiple lines of evidence demonstrate that eukaryotic organisms, including plants, fungi, algae, and animals can produce CH_4_ and N_2_O under aerobic conditions.^3^ Recent advances have revealed that both gases can be generated endogenously in humans through biochemical pathways that do not depend on microbial metabolism.^4^,^5^ Although atmospheric CH_4_ and N_2_O concentrations have increased substantially since preindustrial times, no studies have demonstrated that these ambient levels directly influence human physiology. Instead, current evidence points to endogenous, metabolically regulated formation as the biologically relevant pathway. However, whether rising atmospheric concentrations of these gases may also have subtle or long-term effects on human physiology remains an open question and warrants future investigation. Distinguishing between these pathways emphasizes that the endogenous CH_4_ and N_2_O pathways discussed are chemically distinct from canonical environmental processes and appear closely linked to cellular redox chemistry. Their discovery opens an unexpected bridge between Earth and Life sciences: climate-relevant gases may also act as bioactive molecules and potential biomarkers in human physiology. In this context, CH_4_ and N_2_O emerge not merely as passive trace gases, but as potential reporters, and perhaps modulators, of oxidative and nitrosative stress (**Figure 1**).

**From Earth to cell – rethinking methane formation:** Recent work suggests that CH_4_ production may be a universal feature of life, occurring across virtually all organisms through a reactive oxygen species-driven mechanism.^3^ In metabolically active cells, oxygen metabolism inevitably generates reactive oxygen species, including superoxide anion, hydrogen peroxide, and hydroxyl radicals. In the presence of iron, these species participate in the Fenton reaction, in which reduced iron (Fe²^+^) reacts with hydrogen peroxide to form highly reactive ferryl species and additional hydroxyl radicals. These hydroxyl radicals can cleave a methyl group from methylated sulfur- and nitrogen-containing compounds such as methionine, dimethyl sulfoxide, or methylated amines. The liberated methyl radical then combines with a hydrogen atom to yield CH_4_. This chemistry, validated across diverse eukaryotic taxa, outlines a chemically plausible and experimentally supported pathway for non-microbial CH_4_ formation under aerobic conditions.

In humans, this pathway has been confirmed using stable isotope tracer experiments. Administration of ²H- or ¹³C-labeled dimethyl sulfoxide and ¹³C-methionine, via skin application, oral intake, or direct introduction into blood, resulted in the appearance of isotopically labeled CH_4_ in exhaled breath within minutes.^4^ These results provide unambiguous evidence for radical-driven CH_4_ formation in vivo, independent of gut methanogens.

Far from being a biochemical curiosity, this mechanism sits squarely within the framework of oxidative stress biology. Conditions that elevate reactive oxygen species production, such as acute inflammation, intense physical exertion, hypoxia–reoxygenation injury, or chronic disease, are likely to increase CH_4_ output, provided suitable methylated precursors are present.^6^ The requirement for both oxidative stress and precursor availability may underlie the marked short-term variability in CH_4_ levels observed within individuals without significant gut methanogen populations.

**Methane bioactivity – from inert gas to physiological modulator:** For decades, CH_4_ was considered physiologically inert, a view shaped by its chemical stability and apparent lack of reactivity under normal biological conditions. Over the past 20 years, however, this perception has shifted markedly as experimental evidence has revealed bioactive properties of CH_4_ in oxidative stress and inflammatory settings.^7^ In animal models of ischemia–reperfusion injury affecting the heart, liver, and brain, inhalation of non-asphyxiating concentrations of CH_4_ reduced lipid peroxidation, preserved mitochondrial integrity, and limited apoptotic cell death. Similar protective effects have been observed in models of pulmonary injury, where CH_4_ has been shown to ameliorate tissue barrier function and mitigate the biochemical and structural consequences of oxidative and nitrosative stress.^7^ These findings raise the broader question of whether CH_4_ plays a physiological role in aerobic life beyond being a passive metabolic byproduct.

Although no endogenous biochemical degradation pathway for CH_4_ has yet been identified in aerobic organisms, multiple lines of evidence show that exogenous CH_4_ can influence key regulatory nodes of inflammation and stress adaptation. Notably, CH_4_ modulates master transcriptional switches, affects mitochondrial respiratory capacity, and impacts components of endoplasmic-reticulum–mitochondria-associated pro-apoptotic signaling pathways.^8^ It may also interact with other biologically active gases such as nitric oxide (NO), carbon monoxide, and hydrogen sulfide, suggesting a potential role as part of an interconnected gasotransmitter network.^7^

Within this framework, cells may have evolved mechanisms to sense or utilize CH_4_ as a signaling cue to initiate adaptive stress responses, providing a rationale for its exploration as a therapeutic gas. The therapeutic potential of CH_4_ is already well characterized in several clinically relevant contexts. For example, during veno-venous extracorporeal membrane oxygenation, a life-saving intervention for severe pulmonary failure, CH_4_-enriched gas mixtures have shown promise in mitigating lung injury associated with artificial gas exchange. In experimental heart transplantation, cold storage of donor organs in CH_4_-enriched preservation solution conferred significant protection against endoplasmic reticulum stress-linked pro-apoptotic signaling, mitochondrial damage, and post-transplant cardiac dysfunction.

**Breath methane dynamics and immune responses:** If CH_4_ reflects oxidative stress, its dynamic measurement may also provide insight into immune states. Longitudinal breath monitoring in volunteers has shown both increases and decreases from baseline CH_4_ levels during immune events such as infections and vaccinations.^9^ These shifts align with oxidative degradation or production pathways, as revealed by changes in isotopic CH_4_ signatures. Together, these findings suggest that breath CH_4_ represents a promising, non-invasive marker of immune activation, inflammation, and recovery. A particularly striking observation is the degree of fluctuation within single individuals. Even in people lacking significant gut methanogen populations, CH_4_ output can vary in response to physiological stressors. During acute viral infection, for example, breath CH_4_ may rise sharply within 24 hours, possibly reflecting enhanced reactive oxygen species activity in inflamed tissues. Conversely, after vaccination, CH_4_ levels may also fall, sometimes with accompanying isotopic shifts indicative of oxidative CH_4_ consumption *in vivo*. Such patterns demonstrate that breath CH_4_ is not merely a static marker of microbial activity but a dynamic indicator of systemic redox balance.

**Nitrous oxide – from soils to the human body:** Compared with CH_4_, research on endogenous N_2_O production in humans is far less developed.^10^ Traditionally regarded in environmental science as a microbial product of soil processes and in medicine primarily as an anesthetic and analgesic, N_2_O was until recently rarely considered part of human metabolism. Emerging evidence now suggests that humans can generate N_2_O internally through the nitrate–nitrite–NO pathway. Under conditions of oxidative or nitrosative stress, these reactions may branch toward N_2_O formation. Proposed mechanisms include the reaction of NO with reactive oxygen or nitrogen species, yielding N_2_O as a byproduct, and metal-catalyzed disproportionation of nitrite in acidic or hypoxic environments.^11^ Both scenarios are consistent with N_2_O being formed in parallel with NO when redox balance is disturbed. In addition, NO can be oxidized back to nitrate by hemoglobin, establishing a recycling loop that reinforces endogenous NO and N_2_O formation from nitrate precursors.^12^

Isotope tracer studies provide direct evidence: ingestion of ¹^5^N-labeled nitrate leads to rapid appearance of labeled N_2_O in exhaled breath, confirming the incorporation of dietary nitrogen into N_2_O in vivo. Endogenously formed N_2_O may thus act as a byproduct of nitrosative stress and a potential marker of such conditions. Because the nitrate–nitrite–NO pathway is central to vascular regulation, changes in N_2_O output may also reflect endothelial function or NO bioavailability. Moreover, given NO’s role in immune signaling, N_2_O levels could correlate with immune activation states. Although breath concentrations are typically low, sensitive detection techniques, including stable isotope approaches, can capture diagnostically relevant fluctuations and may also allow differentiation between microbial and stress-related N_2_O formation.

**From environmental chemistry to medical diagnostics:** The recognition that CH_4_ and N_2_O are endogenously produced in the human body bridges two fields that have rarely intersected: atmospheric science and clinical medicine. Analytical techniques originally developed to study trace gases in the atmosphere, such as high-resolution spectroscopy and isotope ratio mass spectrometry, are now being applied to breath analysis. Stable isotope tracing is particularly powerful, allowing researchers to distinguish environmental and microbial sources from endogenous biochemical pathways. By labeling dietary or metabolic precursors, in vivo formation routes can be tracked and quantified. This cross-disciplinary approach not only deepens mechanistic insight but also opens new avenues for diagnostic applications. The measurement of CH_4_ exhaled through the lungs offers a potential means of detecting conditions associated with reduced cardiac output, including heart failure and hemorrhagic shock, together with their gastrointestinal consequences, as well as postoperative bleeding that is often difficult to identify clinically. Despite promising validation in both small and large animal models, several obstacles remain before this approach can be translated into clinical diagnostics. A key limitation is the extremely low baseline concentration of exhaled CH_4_, often approaching the lower detection threshold. In addition, the dynamics of CH_4_ excretion are subject to a “two-gatekeeper” effect, in which variations depend not only on intestinal circulation (inflow) but also on pulmonary function (outflow). Parallel assessment of exhaled CO_2_ may provide complementary information on lung performance.

**From bioactivity to biomarkers:** Recognizing that CH_4_ and N_2_O are not merely metabolic byproducts but may actively participate in physiological processes shifts the discussion from basic biochemistry to clinical potential. Their close links to oxidative and nitrosative stress, together with the ability to measure them rapidly in exhaled breath, make both gases attractive candidates for non-invasive biomarker development. Advances in analytical techniques now allow precise, real-time detection of trace levels and isotopic signatures, providing a unique window into dynamic physiological states. These capabilities bridge mechanistic insight with translational diagnostics and highlight the value of CH_4_ and N_2_O as reporters of systemic redox balance, inflammation, and metabolic stress.

For CH_4_, rapid shifts in breath concentration have been observed during immune activation, inflammation, and metabolic stress. In humans, levels can change within minutes of oxidative challenges, suggesting that CH_4_ may serve as an immediate reporter of redox imbalance. Stable isotope tracing further enhances specificity: administration of ¹³C- or ²H-labeled precursors distinguishes radical-driven CH_4_ production from microbial contributions, aiding differentiation between gut-derived and systemic sources.

N_2_O shows similar promise. As part of the nitrate–nitrite–NO pathway, endogenous N_2_O may reflect vascular and nitrosative stress. Breath measurements could complement established NO assays by revealing additional layers of nitrogen redox chemistry. Isotope-labeled nitrate feeding studies confirm this potential and may help separate stress-related from microbial formation. A particularly compelling application is the detection of gastrointestinal ischemia, a serious but often overlooked complication of circulatory disorders. Preliminary findings suggest that intestinally derived CH_4_ and N_2_O in breath correlate with mesenteric microcirculatory status, pointing to a novel, non-invasive diagnostic approach.

More broadly, the volatility and rapid turnover of CH_4_ and N_2_O make them well suited for longitudinal monitoring. Compared with traditional biomarkers of oxidative and nitrosative stress, such as lipid peroxidation or protein nitration products, which require invasive sampling and typically reflect delayed downstream damage, CH_4_ and N_2_O offer several conceptual advantages. As endogenous gases, they can be measured noninvasively and repeatedly in exhaled breath, enabling real-time monitoring of dynamic physiological responses. Since CH_4_ formation is associated with reactive oxygen species-related chemistry and N_2_O formation to reactive nitrogen species-associated NO pathways, their combined assessment may provide a broader and more discriminative picture of systemic redox status than conventional biomarkers. Unlike many blood-based markers, breath analysis can provide immediate feedback and enable repeated sampling, an advantage in critical care, perioperative monitoring, or during extracorporeal membrane oxygenation. Clinical translation, however, requires systematic studies to establish baseline ranges, variability, and the influence of diet, microbiota, and environment. Determining sensitivity and specificity for distinct pathophysiological states is equally crucial. Nevertheless, the convergence of mechanistic insight, analytical capability, and clinical relevance positions CH_4_ and N_2_O as promising additions to the biomarker toolbox of modern medicine.

**From discovery to clinical practice – opportunities, challenges, and the road ahead:** The recognition that humans generate CH_4_ and N_2_O through non-microbial pathways opens new avenues for understanding redox biology and its links to health and disease. Once viewed solely as environmental actors, these gases are now emerging as biomarkers of oxidative and nitrosative stress accessible through relatively simple, non-invasive breath measurements (**Figure 1**). Clinical applications could be wide-ranging. Inflammatory disorders such as inflammatory bowel disease or rheumatoid arthritis might be monitored more dynamically through breath profiles than with traditional blood markers. In critical care, continuous online monitoring could provide early warning of redox imbalances during surgery, sepsis, or circulatory disturbances such as gastrointestinal ischemia. Even in preventive medicine, individual baseline monitoring could detect subtle physiological shifts before symptoms arise. CH_4_ and N_2_O may also serve as therapeutic guides: if interventions such as antioxidant therapy, dietary nitrate modulation, or iron chelation alter gas output in predictable ways, breath analysis could provide real-time feedback for treatment optimization.

Although CH_4_ output is linked to several gastrointestinal disease phenotypes and nitrosative markers are well documented in inflammatory and cardiovascular diseases, direct disease-specific validation of breath CH_4_, and especially breath or serum N_2_O, as clinical biomarkers is still lacking. Therefore, we present CH_4_ and nitrosative gas readouts as promising but unvalidated markers that require targeted cross-sectional, longitudinal, or interventional studies before diagnostic specificity can be established.

Important challenges remain before clinical adoption. Disentangling microbial from non-microbial contributions, especially CH_4_ in the gut and N_2_O in the oral cavity and gut, is critical. Nevertheless, distinguishing between microbial and non-microbial sources in clinical cohorts will require specific analytical approaches and additional validation studies. Standardized protocols for breath sampling and analysis are needed to ensure reproducibility across laboratories, and large-scale studies must establish baseline ranges, variability, and pathological thresholds.

Addressing these challenges will require strong cross-disciplinary collaboration. Chemists, physiologists, clinicians, and atmospheric scientists each bring essential expertise. By integrating mechanistic insight with advanced analytics and clinical research, CH_4_ and N_2_O may be transformed from perceived greenhouse gases into vital components of the biomarker and therapeutic toolkit.

*During the preparation of this work, the authors used ChatGPT version 5.1 in order to rephrase the text. After using this tool/service, the authors reviewed and edited the content as needed and take full responsibility for the content of its publication.*

*This work was supported by the HU-Rizont call (No. 2024-1.2.3-HU-RIZONT-2024-00033) (to MB and FK).*
